# Supplementary material for: Comparative genomics provides new insights into the diversity, physiology, and sexuality of the only industrially exploited tremellomycete: Phaffia rhodozyma
Source: BMC Genomics. 2016 Nov 9;17:901. doi: 10.1186/s12864-016-3244-7 (PMC5103461; doi:10.1186/s12864-016-3244-7)
Supplement: Additional file 6: — List of orphan genes with links to PFAM (related to Additional file 1: Table S1). (ZIP 1428 kb) [file 12864_2016_3244_MOESM6_ESM.zip › PFAM_UNIQUE/G03270_P.html]

#                                                                            --- full sequence --- -------------- this domain -------------   hmm coord   ali coord   env coord
  
# target name        accession   tlen query name           accession   qlen   E-value  score  bias   #  of  c-Evalue  i-Evalue  score  bias  from    to  from    to  from    to  acc description of target
  
#------------------- ---------- ----- -------------------- ---------- ----- --------- ------ ----- --- --- --------- --------- ------ ----- ----- ----- ----- ----- ----- ----- ---- ---------------------
  
G03270\_P             -            708 PRP1\_N               PF06424.7    133       1.3    8.0   6.3   1   2      0.06        45    3.0   0.7    58    94    75   110    39   121 0.54 - PRP1 splicing factor, N-terminal  
G03270\_P             -            708 PRP1\_N               PF06424.7    133       1.3    8.0   6.3   2   2    0.0037       2.8    7.0   0.6    54   122   635   700   575   705 0.82 - PRP1 splicing factor, N-terminal  
G03270\_P             -            708 SOBP                 PF15279.1    306       1.5    7.8   6.5   1   2       8.5   1.1e+03   -1.7   0.1   148   148    97    97    13   180 0.41 - Sine oculis-binding protein  
G03270\_P             -            708 SOBP                 PF15279.1    306       1.5    7.8   6.5   2   2     0.002      0.27   10.2   2.3    64   260   211   443   182   464 0.62 - Sine oculis-binding protein
